# Evaluating the psychometric properties of three WHO instruments to assess knowledge about human rights, attitudes toward persons with mental health conditions and psychosocial disabilities, and practices related to substitute decision-making and coercion in mental health

**DOI:** 10.3389/fpsyt.2024.1435608

**Published:** 2024-09-06

**Authors:** Maria Francesca Moro, Leveana Gyimah, Ezra Susser, Joana Ansong, Jeremy Kane, Caroline Amissah, Oye Gureje, Akwasi Osei, Andrea Norcini Pala, Dan Taylor, Nathalie Drew, Humphrey Kofie, Florence Baingana, Sally-ann Ohene, Nii Lartey Addico, Abdul Fatawu, Michela Atzeni, Silvia D’Oca, Mauro Giovanni Carta, Michelle Funk

**Affiliations:** ^1^ Department of Epidemiology, Mailman School of Public Health, Columbia University, New York, NY, United States; ^2^ Policy, Law and Human Rights, Department of Mental Health and Substance Use, World Health Organization, Geneva, Switzerland; ^3^ World Health Organization (WHO) Country Office for Ghana, Accra, Ghana; ^4^ New York State Psychiatric Institute, New York, NY, United States; ^5^ Mental Health Authority, Ghana Ministry of Health, Accra, Ghana; ^6^ World Health Organization (WHO) Collaborating Centre for Research and Training in Mental Health, Neurosciences and Substance Abuse, Department of Psychiatry, University of Ibadan, Ibadan, Nigeria; ^7^ Department of Community Health Sciences, State University of New York (SUNY) Downstate, Brooklyn, NY, United States; ^8^ MindFreedom Ghana, Accra, Ghana; ^9^ Mental Health Society of Ghana, Accra, Ghana; ^10^ Mental Health and Substance Abuse, World Health Organization (WHO) Regional Office for Africa, Brazzaville, Republic of Congo; ^11^ Department of Medical Sciences and Public Health, University of Cagliari, Cagliari, Italy

**Keywords:** mental health, human rights, psychometric properties, knowledge, attitudes, practices, validity, reliability

## Abstract

**Background:**

Instruments to assess the knowledge about the rights of persons with mental health conditions and psychosocial disabilities, the attitudes toward their role as rights holders, and mental health professionals’ practices related to substitute decision-making and coercion are either missing or lack evaluation of their validity and reliability.

**Aim:**

The aim of this study is to evaluate the validity and reliability of three instruments developed to fill this gap in the literature, the World Health Organization’s QualityRights (WHO QR) Knowledge questionnaire, the WHO QR Attitudes questionnaire, and the WHO QR Practices questionnaire.

**Methods:**

A sample of participants was recruited and completed an online survey. Content validity and face validity were assessed for the three questionnaires. Based on the characteristics of the questionnaires, different approaches were used to assess their construct validity (confirmatory factor analysis, known group validity, and convergent and divergent validity). Internal consistency was evaluated using Cronbach’s alpha and test re-test reliability using Pearson’s and Spearman’s r coefficients.

**Results:**

The analyses conducted indicate that the three questionnaires are valid and reliable instruments to evaluate the knowledge about the rights of persons with mental health conditions and psychosocial disabilities, the attitudes toward their role as rights holders, and mental health professionals’ practices related to substitute decision-making and coercion.

**Conclusion:**

This finding lends support to the use of these instruments both within mental health services and in the general population for a better understanding of current knowledge, attitudes, and practices related to a human rights–based approach to mental health in mental health services and the community.

## Introduction

People with mental health conditions and psychosocial disabilities are commonly exposed to human rights violations both within the general community and the mental health care system (1–5). The negative consequences of these violations on the health of persons with mental health conditions and psychosocial disabilities have been widely documented but attempts to improve the situation have met with little success. Among the barriers to addressing this problem, three are particularly important: 1) the general public and mental health professionals’ lack of knowledge about human rights (6, 7); 2) the negative attitudes toward people with mental health conditions or psychosocial disabilities’ role as right holders (i.e., their role as persons entitled to exercise all human rights on an equal basis with others) (8–11); and 3) the negative practices implemented in mental health services, such as the use of seclusion, restraint, and substitute decision-making approaches to care that keep people with mental health conditions and psychosocial disabilities disempowered and contribute to the negative image that the general public holds of them (3, 4, 12).

Education on human rights may have a fundamental role in changing negative attitudes and, in turn, practices and behaviors leading to human rights violations. The Knowledge-Attitude-Practice (KAP) framework, also found in literature as Knowledge-Attitude-Behavior framework (13, 14), helps explain this relationship. According to the KAP framework, the accumulation of knowledge about a particular issue leads to changes in the predisposition to respond (the attitude). Over time, this causes a change in practice that is in agreement with the attitude (13). The legitimacy of the KAP approach in the health field, although not entirely free from criticism (15–17), is largely supported by the scientific literature (13, 18–20) and has formed the basis for many successful public health interventions (21–25). Within this article context, according to the KAP framework, improved knowledge of human rights and how to apply rights-based, person-centered approaches among mental health professionals and other stakeholders would lead to positive attitudinal changes toward people with mental health conditions or psychosocial disabilities as rights holders. This would also lead, over time, to reduced human rights violations and improved practices consistent with a human rights–based approach.

The KAP approach has rarely been implemented in its entirety within the mental health and human rights field. There have been numerous initiatives aimed to either increase awareness about human rights (26–31) or challenge the negative attitudes of society toward people with mental health conditions and psychosocial disabilities (32–35). However, only a few initiatives to increase knowledge and change attitudes have also evaluated a change in practices. For instance, among the studies to address mental health professionals’ stigma, only two (36, 37) assessed a general change in practices.

In parallel with these initiatives, many instruments to measure knowledge and attitudes have been developed (31, 38, 39), whereas instruments assessing a change in practices are extremely rare because this aspect is neglected in research. However, even the instruments developed to date present important limitations. The majority of these instruments have not been tested for reliability and validity. This is an issue because the use of instruments that lack evidence of validity and reliability may lead to information bias. Additionally, these instruments often do not follow the human rights approach and language promoted by international human rights treaties such as the United Nations Convention on the Rights of Persons with Disabilities (UN CRPD). The existing instruments were mainly developed by health practitioners and often reflect their position, even when this is in contradiction with the perspective of people with mental health conditions and psychosocial disabilities and their organizations.

As far as we know, there are no validated instruments to specifically evaluate the knowledge regarding persons with mental health conditions and psychosocial disabilities’ human rights included under the UN CRPD. There are several validated instruments to assess negative attitudes toward this group, but none of these instruments focus specifically on attitudes toward their role as rights-holders, although such aspect is fundamental to achieve full participation in society (38). Furthermore, there are no instruments to assess changes in practices related to substitute decision-making and coercion, although such human rights violations are common in mental health services. Instruments able to capture these aspects of practice change are much needed.

The present paper aims to fill this gap by evaluating the validity and reliability of three World Health Organization instruments developed to assess the knowledge about the rights of persons with mental health conditions and psychosocial disabilities, the attitudes toward their role as rights holders, and mental health professionals’ practices related to substitute decision-making and coercion. The study was carried out in Ghana, a country that recently came under scrutiny for human rights violations in mental health and where several interventions are ongoing to tackle these violations (40). The instruments under evaluation could be useful in evaluating some of these initiatives. Indeed, in a further study, we have used them in evaluating change in practices in a Randomized Controlled Trial (RCT) in Ghana, with results forthcoming in a subsequent paper.

## Materials and methods

### Study setting

The study was conducted in Ghana as part of the project funded by the European Commission (EIDHR 2018–400431) “Empowering persons with psychosocial disabilities to fight for their rights: An implementation of the CRPD and QualityRights principles in Ghana, Lebanon, and Armenia.” Prior to this study, several local and international human rights organizations and scientific publications reported that violations of the rights of persons with mental health conditions and psychosocial disabilities are common in psychiatric facilities and the general community in Ghana (41–45).

### Ethics approval and informed consent

The study was approved by the Ghana Health Services Ethics Review Committee (study protocol approval: GSH-ERC 005/01/21. It was conducted according to the Declaration of Helsinki and its revisions. All data were made confidential according to the provisions that protect privacy in Ghana (Data Protection Act, 2012). Participants were required to give informed consent electronically at the recruitment and the second administration of the instruments after 10 days. In this study, as required by the UN CRPD, individuals’ right to legal capacity (including the right to provide informed consent) was not denied on the basis of disability status.

### Description of the instruments to evaluate

Three self-report instruments were evaluated in this study:

- The World Health Organization’s QualityRights Knowledge questionnaire (WHO QR Knowledge) measures knowledge about the rights of persons with psychosocial disabilities and mental health conditions included under the UN CRPD. This instrument includes questions around the meaning of different rights (e.g., “Informed consent is when a person’s family members receive information about different possible treatment options in order to make an informed decision”), their application and/or violation (e.g., “Human rights can never be restricted” and “In supported decision-making, support should increase throughout life”) and states’ obligations (e.g., “The Convention on the Rights of Persons with Disabilities is binding on countries which have ratified it”). The items are dichotomous (respondents are required to judge whether the statements are “True” or “False”), with correct responses scoring one point and incorrect responses scoring zero points. Scores on the WHO QR Knowledge questionnaire are calculated by summing up responses across items for each participant as per standard practice. A high score on the questionnaire indicates a high level of knowledge about the UN CRPD principles. More details on the WHO QR Knowledge are provided in **Supplementary Table 1** (original version) and **Table 1** (final version).
- The World Health Organization’s QualityRights Attitudes questionnaire (WHO QR Attitudes) measures attitudes toward people with psychosocial disabilities or mental health conditions as rights-holders. This instrument comprises items organized in three subscales: Subscale 1 includes questions on the attitudes toward mental health services approach (e.g., “People with dementia should always live in group homes where staff can take care of them” and “The service environment has little to do with people’s mental health and well-being”); Subscale 2 includes questions on the attitudes toward involuntary and coercive practices (e.g., “Controlling people using mental health services is necessary to maintain order” and “The use of seclusion and restraint is needed if people using mental health services become threatening”); and Subscale 3 includes questions on the attitudes toward people with psychosocial disabilities or mental health conditions as decision-makers and full members of society (e.g., “People with psychosocial disabilities/mental health conditions should not be hired in work requiring direct contact with the public” and “Persons with mental health conditions should not be given important responsibilities”). Answers are provided on a five-point Likert scale (“Strongly disagree,” “Disagree,” “Neutral,” “Agree,” and “Strongly agree”) and rated following this scheme: “Strongly disagree” is scored as one point, “Disagree” as two points, “Neutral” as three points, “Agree” as four points, and “Strongly agree” as five points. All items are negatively worded, except for items 7, 13, and 17 (which require reverse scoring). Scores on the questionnaire are calculated by summing responses across each participant’s items. Because the items are ordinal and have at least five categories, they are treated as an ordinal approximation of a continuous variable in the analyses (46, 47). High scores on the questionnaire indicate negative attitudes toward people with psychosocial disabilities’ role as rights-holders. More details on the WHO QR Attitudes are provided in **Table 2** and **Supplementary Table 2**.
- The World Health Organization’s QualityRights Practices questionnaire (WHO QR Practices) measures the practices related to substitute decision-making and coercion in mental health units. This instrument is divided in two subscales. The first subscale measures how frequently the respondent has used practices such as seclusion or restraints in the last 3 months and includes questions such as “I prescribed or administered a treatment although the service user did not want it” or “I yelled or used verbal aggression to get service users to comply with requests.” The answers are provided on a seven-point Likert scale, rated following this scheme: “Never” is scored as one point, “A few times in the last 3 months” as two points, “Once a month or less” as three points, “A few times a month” as four points, “Once a week” as five points, “A few times a week” as six points, and “Every day” as seven points. Items 4 and 5 need to be reverse-scored. A high score indicates practices related to substitute decision-making and coercion are used frequently by the respondent. The second subscale assesses the level of agreement between the respondents and the mental health professionals working in their units in the use of practices related to substitute decision making and coercion. It includes questions such as “Mental health professionals in my unit use restraint to control unsettled situations in the ward” or “Mental health professionals in my unit use seclusion and chemical restraints.” Answers are provided on a five-point Likert scale, rated as follows: “A lot less than me” is scored as one point, “Less than me” as two points, “As much as me” as three points, “More than me” as four points, and “A lot more than me” as five points. Item 11 needs to be reverse-scored. High scores indicate that the mental health professionals working in the units of the respondents are more willing to use practices related to substitute decision-making and coercion than the respondents are. Because the items are ordinal and have at least five categories, they are treated as an ordinal approximation of a continuous variable in the analyses (46, 47). More details on the WHO QR Practices are provided in **Supplementary Table 3** (original version) and **Table 3** (final version).

**Table 1:** World Health Organization’s QualityRights Knowledge questionnaire (WHO QR Knowledge): Final version with 24 items.

Please indicate if the following statements are True (T) or False (F)			
		T	F
**1**	The Universal Declaration of Human Rights (UDHR) is a law.		
**2**	Violations of human rights can only be carried out by individuals not by governments.		
**3**	The Convention on the Rights of People with Disabilities (CRPD) is a convention that protects the rights of all marginalized groups.		
**4**	According to the CRPD, people with dementia have the right to live in the community and to choose their living arrangements.		
**5**	Informed consent is when a person’s family members receive information about different possible treatment options in order to make an informed decision.		
**6**	Advance plans/directives are documents made by health practitioners to plan in advance the treatment of people using the service.		
**7**	To promote legal capacity, family members, caregivers, and supporters should help people make decisions by explaining different options but should not assist in communicating decisions to others.		
The Convention on the Rights of People with Disabilities:			
**8**	Was intended to create new rights for people with disabilities		
**9**	Adopts the medical and charity models of disability		
According to the human rights model, people diagnosed, perceived or self-identifying as having a mental health condition, psychosocial, intellectual or cognitive disability:			
**10**	Must show their ability to understand rights in order to claim them		
**11**	Have the right to have attitudinal and environmental barriers removed		
**12**	Need treatment to “fix” or heal them		
The right to liberty and security of a person in the CRPD means that:			
**13**	Mental health laws can authorize people to be detained if they are diagnosed with a mental health condition and if they are perceived as dangerous.		
In supported decision-making, support:			
**14**	Can be declined by the person		
**15**	Should concern only complex decisions		
**16**	Should increase throughout life		
Forced treatment, seclusion, and restraint:			
**17**	Keep people safe		
**18**	Are forms of coercion		
**19**	Improve recovery if used correctly		
**20**	Can cause harm		
Which of the following promote the right to legal capacity?			
**21**	Healthcare provider-led recovery and/or treatment plans		
**22**	Substitute decision-making		
According to the Convention on the Rights of Persons with Disabilities:			
**23**	The use of restraints is only allowed in cases where the service lacks adequate human resources.		
**24**	There is no need to make laws to protect people with disabilities from exploitation, violence, and abuse because the CRPD already ensures their protection.		

**Table 2:** World Health Organization’s QualityRights Attitudes questionnaire (WHO QR Attitudes).

		Strongly disagree	Disagree	Neutral	Agree	Strongly agree
1	Nothing can be improved within mental health services without additional resources.					
2	The service environment has little to do with people’s mental health and well-being.					
3	People with dementia should always live in group homes where staff can take care of them.					
4	People with psychosocial disabilities/mental health conditions should not be hired in work requiring direct contact with the public.					
5	Taking medication is the most important factor to help people with mental health conditions get better.					
6	You can only inspire hope once a person is no longer experiencing symptoms.					
7	People using mental health services should be empowered to make their own decisions about their treatment.					
8	Following advice of other people who have experienced mental health issues is too risky.					
9	The opinions of health practitioners about care and treatment should carry more weight than those of a person with an intellectual disability.					
10	It is acceptable to pressure people using mental health services to take treatment that they don't want.					
11	Persons with mental health conditions should not be given important responsibilities.					
12	When people experience a crisis, health practitioners or families should make decisions based on their ideas about what is best for them.					
13	People with intellectual disabilities have the right to make their own decisions, even if I do not agree with them					
14	Controlling people using mental health services is necessary to maintain order					
15	The use of seclusion and restraint is needed if people using mental health services become threatening					
16	People at risk of harming themselves or others should be isolated in a locked room.					
17	Involuntary admission does more harm than good.					

**Table 3:** World Health Organization’s QualityRights Practices questionnaire (WHO QR Practices): Final version.

Please indicate how many times in the last three months you used the following strategies within your psychiatric unit. Select only one option for each statement.								
		Every day	A few times a week	Once a week	A few times a month	Once a month or less	A few times in the last 3 months	Never
1	**I used seclusion** (for instance, ordering or keeping service users in a locked room).							
2	**I used physical restraints** (for instance, using ties or other mechanical devices to restrain service users).							
3	**I prescribed or administered a treatment although the service user did not want it.**							
4	**I used chemical restraints** (for instance, prescribing or administering an injection to calm the behavior of service users without their consent).							
5	**I yelled or used verbal aggression to get service users to comply with requests.**							
For each statement, mark the box that most accurately reflects your response.								
		A lot less than me	Less than me	As much as me	More than me	A lot more than me		
6	Mental health professionals in my unit use seclusion and physical/chemical restraints.							
7	Mental health professionals in my unit yell or use verbal aggression to get service users to comply with requests.							
8	Mental health professionals in my unit prescribe or administer treatments to control the behavior of service users.							
9	Mental health professionals in my unit use restraints to control unsettled situations in the ward.							

### Instruments development, testing, and piloting

The WHO QualityRights research team developed the World Health Organization’s QualityRights Knowledge (WHO QR Knowledge) questionnaire and the World Health Organization QualityRights Attitudes questionnaire (WHO QR Attitudes) after a long process of discussion and consultation with different stakeholders (e.g., organizations of persons with mental health conditions and psychosocial disabilities, mental health professionals, human rights advocates, academics, experts in psychometrics, and members of the government) from a variety of countries. The research team of the project “Empowering persons with psychosocial disabilities to fight for their rights: An implementation of the CRPD and QualityRights principles in Ghana, Lebanon, and Armenia” developed the WHO QualityRights Practices questionnaire (WHO QR Practices).

First, the research teams reviewed the literature searching for pre-existing instruments to assess knowledge about disability rights, attitudes toward people with psychosocial disabilities and mental health conditions, and practices related to substitute decision-making and coercion. Then, the research teams developed a first draft of the questionnaires. A group of stakeholders (e.g., organizations of persons with mental health conditions and psychosocial disabilities, mental health professionals, human rights advocates, academics, experts in psychometrics, and members of the government) provided feedback on the drafts, and revisions were incorporated. A second group of stakeholders from different countries then reviewed the revised questionnaires. The final versions of the questionnaires were used in the present study.

### Collection of data

#### Sociodemographic variables

In this study, the questionnaire used to collect data included the following sociodemographic variables: gender; age in years; educational attainment; “main” background (with possibility to select only one option among: person with psychosocial, intellectual, and cognitive disability or mental health condition; person with other disabilities; family member or care partner; mental health or related practitioner; health practitioner; lawyer; human rights advocate; policymaker/analyst; academia; other); region of residence; main language; family member(s) with a psychosocial disability or mental health condition; and identification as “person with a psychosocial disability or mental health condition.”

#### Additional instruments used

The following instruments were administered with the WHO QR Attitudes questionnaire for evaluating convergent and divergent validity:

- The Community Attitudes Toward the Mentally Ill (CAMI) (48) is a 40-item, self-report instrument to assess the public’s attitudes toward persons with mental health conditions. There are four subscales within the CAMI: Authoritarianism, Benevolence, Social restrictiveness, and Community mental health ideology. High scores on the CAMI subscales indicate endorsement of authoritarianism, benevolence, social restrictiveness, and community mental health ideology. This instrument has been used in a previous study in Ghana (49) and has shown acceptable reliability, with Cronbach’s alpha values ranging between 0.71 and 0.75 for all the subscales, except for the authoritarianism subscale, for which Cronbach’s alpha was 0.31 (likely because the authors did not include two items from the original scale, whereas we included these items in the present study). It was hypothesized that higher scores on the WHO QR Attitudes (showing negative attitudes) would be associated with higher scores on the Authoritarianism and Social Restriction subscales and lower scores on the Benevolence and Community Mental Health Ideology (CAMI) subscales.
- The Attribution questionnaire (AQ) (50) is a 27-item, self-report instrument to measure stigma toward persons with mental health conditions. The AQ consists of a brief vignette about “Harry,” a person with a diagnosis of schizophrenia, followed by questions organized in nine subscales assessing nine constructs: Blame, Anger, Pity, Help, Dangerousness, Fear, Avoidance, Segregation, and Coercion. High scores on the AQ subscales indicate a high endorsement of the corresponding constructs. We hypothesized that higher scores on the WHO QR Attitudes (showing negative attitudes) would be associated with higher scores on the Blame, Anger, Pity, Dangerousness, Fear, Avoidance, Segregation, and Coercion subscales and lower scores on the Help subscale.

#### Procedure for data collection

All data were collected through an online platform. Participants received a first email with a link to a survey requesting the sociodemographic variables, the WHO QR Knowledge, the WHO QR Attitudes, the WHO QR Practices, the CAMI, and the AQ. After 10 days, participants received a second email with a link to a survey including only the WHO QR Knowledge, the WHO QR Attitudes, and the WHO QR Practices. These data were used for the evaluation of the test–retest reliability.

### Sample and recruitment procedure

Opportunistic sampling was used to recruit participants among mental health professionals, members of organizations of persons with disabilities, human rights advocates, academics, and government members, aged 18 years or older and able to speak English. Potential participants were identified using three strategies. First, participants were randomly selected from the register of mental health professionals employed in three psychiatric hospitals (Accra, Ankaful, and Pantang) and invited to be screened for eligibility (via email or phone). Second, trained research assistants, who had professional liaisons with local experts on human rights in mental health, identified potential participants among their contacts (mental health professionals, members of organizations of persons with disabilities, human rights advocates, academics, and government members). This strategy guaranteed that this group of potential participants included only experts in human rights in mental health. Third, the trained research assistants used a recently published list of Ghanaian organizations working in the mental health field to identify and contact potential participants. Potential participants were selected among the organizations’ affiliates who were not experts in human rights in mental health. The potential participants selected through the second and third strategies were then invited to participate in the study by email or phone and provided informed consent electronically. The three strategies allowed us to recruit both experts and non-experts in human rights in mental health, including participants who were mental health professionals and participants who were not. This is important because two of the instruments under evaluation aimed ultimately to target a broader audience. Sample size calculations indicated the need to recruit 280 participants to have enough power to perform the analyses.

### Data analytic approach

#### Description of the sample

Descriptive analyses were conducted for the sociodemographic variables. Univariate analyses were used to examine continuous variables by assessing the mean, the median, and the spread of the data. Tabular analyses were used to examine categorical variables by assessing frequencies. Missing data were reported for each variable.

#### Validity

In the present study, content validity, face validity, and construct validity were used to assess the validity of the instruments under investigation.

A given instrument is said to have content validity when its development includes a review of existing data and literature, and an independent panel of experts in the subject matter under investigation (usually seven or more) confirms that the instrument items are relevant and reflect the domain of interest (51, 52). In the present study, the instruments were developed following the first criteria, and, then, content validity was assessed by seven experts in human rights in mental health that examined the instruments to ensure that they were consistent with their underlying conceptual frameworks. Experts also evaluated the performance of the items on four dimensions (item consistency with the content area, item wording clarity, items perceived difficulty, and whether the items should be included in instruments) using a dichotomous response scale (yes vs. no) (53, 54). “Yes” answers were scored as 1 and “No” as 0. The maximum overall score for content validity was 476 for the WHO QR Attitudes (each of the 17 items was evaluated on four dimensions by seven experts, and each dimension could have a maximum score of 1: 17 × 4 × 7 = 476), 1,036 for the WHO QR Knowledge, and 336 for the WHO QR Practices. Based on these scores, average content validity indexes were calculated for all the instruments (dividing the actual overall score for content validity by the maximum overall score for content validity) (55). The recommended content validity index cutoff value of 0.75 was considered acceptable (55). Experts had the possibility to provide comments for each item.

A given instrument is said to have face validity when individuals in the target population agree the instrument appears to measure the dimension under investigation (51). In the present study, face validity was assessed by seven lay stakeholders (including persons with mental health conditions or psychosocial disabilities, members of civil society organizations, and mental health professionals). The lay stakeholders examined the instruments using a dichotomous response scale (yes vs. no) to evaluate if the items were clear, easy to understand, and relevant (53, 54). The maximum overall score for face validity was 357 for the WHO QR Attitudes (each of the 17 items was evaluated on three dimensions by seven lay stakeholders, and each dimension could have a maximum score of 1: 17 × 3 × 7 = 357), 756 for the WHO QR Knowledge, and 252 for the WHO QR Practices. Stakeholders had the possibility to provide comments for each item.

Construct validity assessment requires that a conceptual model of the construct of interest is postulated, and its relationships with other relevant constructs in the subject-matter domain have been described (51, 56). If the assessment results are in agreement with the conceptual model and the relationships postulated for the construct of interest, then the questionnaire is considered valid. Based on the characteristics of the questionnaires, different approaches were used to assess their construct validity.

- For the World Health Organization’s QualityRights Knowledge questionnaire (WHO QR Knowledge):
- Known group validity (i.e., the capacity of a questionnaire to differentiate groups of respondents who may be anticipated to have a different score in a predicted direction) (51) was used to compare the total scores for experts and non-experts in human rights using non-parametric Wilcoxon–Mann–Whitney test. We hypothesized that experts, who have greater knowledge on human rights, would have higher scores on the WHO QR Knowledge than non-experts.
- Confirmatory Factor Analysis (CFA) was used to evaluate the one dimension postulated (i.e., knowledge about human rights). A model was fitted including this dimension and using the estimator Weighted Least Square Mean and Variance adjusted (WLSMV) for dichotomous variables. Goodness of fit indices were used to evaluate this model: Tucker–Lewis Index (TLI) ≥ 0.95 and ≥ 0.90 indicating good and sufficient fit, respectively; Root Mean Square Error of Approximation (RMSEA) ≤ 0.05 or ≤ 0.08 indicating good and sufficient fit, respectively (56). Finally, factor indicators with loadings lower than 0.30 were considered for removal, and path diagrams were used for a visual comparison of the factor loadings.
- For the World Health Organization’s QualityRights Attitudes questionnaire (WHO QR Attitudes):
- Confirmatory Factor Analysis was used to evaluate the three dimensions postulated (i.e., 1) Attitudes toward mental health services approach, 2) attitudes toward involuntary and coercive practices, and 3) attitudes toward people with psychosocial disabilities or mental health conditions as decision-makers and full members of society). This factor structure was evaluated using the same approach described for the factor analysis of the WHO QR Knowledge, but using the estimator MLR for continuous variables, that provides better standard error estimation under deviations from normality (56). Factor correlations were estimated using Pearson’s r coefficient (56).
- Convergent and divergent validity (i.e., the capacity of a questionnaire to have high correlations with measures of related constructs and weak correlations with measures of unrelated constructs) (56) were evaluated calculating Pearson’s correlations between the WHO QR Attitudes, the subscales of the Community Attitudes Toward the Mentally Ill (CAMI), and the subscales of the Attribution Questionnaire (AQ). It was hypothesized that higher scores on the WHO QR Attitudes (showing negative attitudes) would be associated with the following: a) higher scores on the Authoritarianism and Social Restriction subscales (CAMI); b) lower scores on the Benevolence and Community Mental Health Ideology subscales (CAMI); c) higher scores on the Blame, Anger, Pity, Dangerousness, Fear, Avoidance, Segregation, and Coercion subscales (AQ); and d) lower scores on the Help subscale (AQ).
- For the World Health Organization’s QualityRights Practices questionnaire (WHO QR Practices):
- Confirmatory Factor Analysis was used to evaluate the two dimensions postulated (i.e., 1) practices related to substitute decision-making and coercion used by the respondent, and 2) level of agreement between the respondents and the mental health professionals working in their units in the use of practices related to substitute decision-making and coercion). This factor structure was evaluated using the same approach described for the factor analysis of the WHO QR Attitudes.

#### Reliability

In the present study, Cronbach’s alpha was used to assess the internal consistency of the final version of the instruments (after dropping items based on validity assessment) total scales and subscales. Acceptable values of alpha range from 0.70 to 0.95 (57, 58).

Test–retest reliability for the final versions of the instruments was estimated using Pearson’s and Spearman’s r coefficients.

### Sample size and power calculation

Given that factor analyses (i.e., CFAs) were performed for all the questionnaires, we referred to this analysis when calculating the sample size. There is no consensus in the literature regarding what sample size is required when performing a CFA to keep power close to 0.80. The majority of researchers consider 5 to 10 subjects per item to provide sufficient power to conduct a CFA (59, 60). Simulation studies have shown that, when variables are normally distributed, an appropriate sample size for a simple CFA model is n = 150 (61).

However, to obtain a more precise estimate of the sample size to be used in the study, sample size calculations were performed using Monte Carlo simulations. The focus was on the model proposed for the CFA of the WHO QR Attitudes because this was the most complex model examined (and thus, its sample size should be sufficiently powered to perform the other, simpler, CFAs).

In the Monte Carlo simulations, 10,000 replications were used for each analysis to ensure that stability had been reached. The sample size calculation was based on the following criteria: 1) parameter and standard error biases do not exceed 10% for any parameter in the model; 2) standard error bias for one of the parameters for which power is being assessed (i.e., subscale at the questionnaire) does not exceed 5%; 3) coverage remains between 0.91 and 0.98; and 4) power is at least to 0.80 (61). Results were estimated from the three-factor proposed for the aforementioned CFA, assuming the lowest conventionally acceptable correlation coefficient of 0.3 to indicate a positive factor loading. Based on these criteria, we calculated that 280 participants were required.

Factor analyses and sample size calculations were performed with MPLUS 8.5 (62). Reliability analyses were performed with R. All other analyses were performed with SAS 9.4 (SAS Institute, Cary NC).

## Results

### Characteristics of the respondents

Overall, 393 persons agreed to participate of the 788 invited (49.9%). Forty-seven respondents were excluded because, even though they provided informed consent, they did not continue the survey. Twenty-six respondents who completed only the survey questions regarding sociodemographic variables were removed. In total, 320 participants (81.4%) completed at least some of the survey questions from the WHO QR questionnaires and were included in the analyses. There were differences between completers of at least some of the survey questions from the WHO QR questionnaires and “non-completers” (i.e., people who completed only the survey questions on sociodemographic variables) regarding gender (among completers 39.7% were women, 59.7% men, and 0.6%, other gender, whereas, among the 26 non-completers, 57.7% were women and 42.3% men) and median age (among completers, median age was 37, whereas, among the 26 non-completers, median age was 34.5).

More participants completed the WHO QR Attitudes (n = 320) because this was the first questionnaire to be presented in the survey. The WHO QR Knowledge (n = 283) was the second questionnaire. Only mental health professionals were invited to complete the WHO QR Practices (n = 81).

**Supplementary Table 4** shows respondents’ (i.e., completers of at least some of the survey questions from the WHO QR questionnaires) characteristics. A total of 39.7% of participants identified as women, 59.7% as men, and 0.6% as other gender. The median age was 37 (mean of 39.3 and standard deviation of 10.2). A total of 10% of participants had a high school degree or an equivalent title, 10% had some college, 28.1% had a college degree, 27.8% a master’s degree and 7.8% a professional degree (the education level in the country is lower as would be expected due to the participants’ selection criteria). Respondents also reported their “main” background (they could select only one option among the ones proposed): 27.5% were mental health practitioners; 17.8% were other health practitioners; 15.9% were persons with a psychosocial disability or mental health condition; 15.0% were persons with other disabilities; 10.9% were human rights advocates; 4.1% were family members or care partners; and the remaining were academics, lawyers, and policymakers. All the Ghanaian regions (except for North-East) were represented, although most participants were from Greater Accra (51.6%). A total of 40.6% of respondents indicated English as their first language. In addition, 35.3% of the participants reported having a person with a psychosocial disability or mental health condition in their close family and 19.1% identified as a person with a psychosocial disability or mental health condition.

One hundred fifty-four participants answered a second test–retest assessment. A total of 113, 124, and 11 participants completed the WHO QR Knowledge, the WHO QR Attitudes, and the WHO QR Practices, respectively.

### WHO QualityRights knowledge questionnaire

A total of 283 participants completed the WHO QualityRights knowledge questionnaire. There were no significant differences between the total sample (i.e., the 320 participants that completed at least some of the survey questionnaires from the WHO QR questionnaires) and completers of the WHO QR Knowledge questionnaire regarding gender (in the total sample, 39.7% were women, 59.7% men, and 0.6% other gender, whereas, among the WHO QR Knowledge completers, 38.2% were women 61.2%, men and 0.6% other gender) and median age (median age was 37 both in the total sample and among the WHO QR Knowledge completers).

#### Validity

**Supplementary Table 5** shows the content validity assessment performed by the seven experts in human rights in mental health. The first column reports the evaluation of the items’ consistency, the second of the items’ clarity, the third of the items’ difficulty, and the fourth column describes if the item was deemed deserving to be included in the questionnaire. When all seven experts said, for instance, that one item was clear, that item got a score of 7/7. The maximum score for each item was 28. The questionnaire obtained an overall score of 961/1,036 when evaluated for the performance of its items. An average content validity index 0.92 was calculated from this score, indicating that the WHO QR Knowledge items were overall relevant and clear. Item QR19_K received a low score (18/28) and, after discussion with the experts, was removed from the questionnaire. This item was meant to convey the idea that people cannot be detained (in health facilities) because of their disability (that may lead others to worry about their “social dangerousness”). However, it was interpreted as “people with disabilities can be detained (as it happens for people without disabilities) if they met other criteria such as dangerousness,” and, thus, it was removed.

**Supplementary Table 6** shows the face validity assessment performed by seven lay stakeholders. The first column reports the evaluation of the items’ clarity, the second describes if the items were easy to understand, whereas the third reports if the item was deemed relevant. The maximum score for each item was 21. The overall score of the questionnaire was 701/756 and all the items received high scores (ranging from 16 to 21), indicating that the questionnaire was clear, easy to understand, and relevant for the target population.

**Figure 1:**
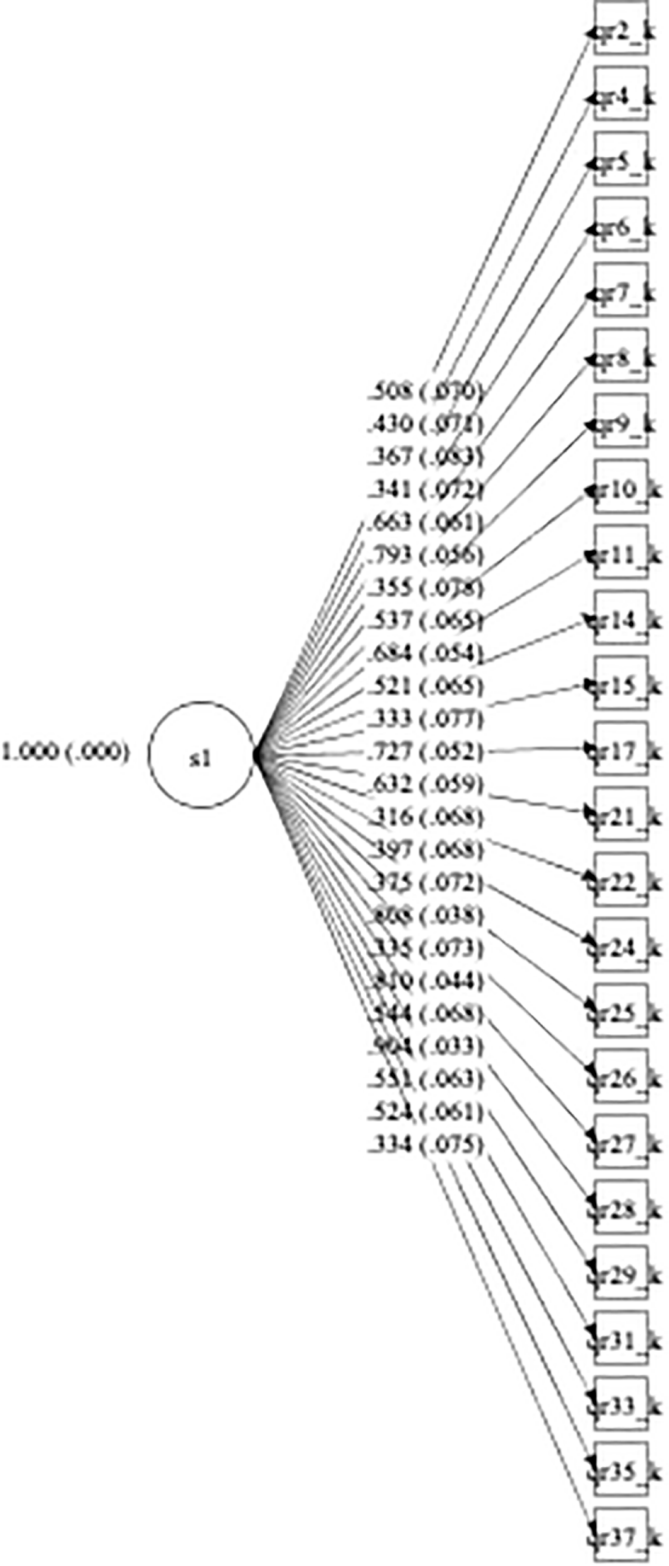
Confirmatory factor analysis path diagram for WHO QR Knowledge (final version). *Standardized loadings (and standard errors).

A Confirmatory Factor Analysis was conducted, fitting a model with one factor as postulated. The items that had factor loadings with values lower than 0.3 (items 1, 3, 12, 13, 16, 20, 23, 30, 32, 34, and 36) or correlated negatively with the underlying factor (items 12 and 18) were excluded from the final model. The final 24-item model showed an acceptable fit (RMSEA = 0.049; CFI = 0.92; TLI = 0.91), and all the factor loadings were supported (see **Figure 1**), with values greater than 0.3 and significant, indicating that the questionnaire is able to measure the construct of interest.

With respect to the known group validity, WHO QR Knowledge scores achieved by both the experts and nonexperts group displayed a non-normal distribution and ranged from 8 to 24 (median = 21, mean = 19.87, Confidence Level (CL) = 18.52–21.22, standard deviation = 3.62, lower quartile = 19, and upper quartile = 23) and 5 to 22 (median = 11, mean = 11.98, CL = 11.46–12.49, standard deviation = 4.15, lower quartile = 9, and upper quartile = 15). Participants in the “experts group” were found to be different from “nonexperts” in the hypothesized direction, with experts having higher scores on the WHO QR Knowledge than non-experts (Z = 7.3838, p-value < 0.0001), indicating that the questionnaire is able to differentiate people with knowledge about the rights of persons with psychosocial disabilities and mental health conditions and people without this knowledge.

#### Reliability

The internal consistency of the final version of the questionnaire, with 24 items, was obtained. The overall Cronbach’s alpha value for the total scale indicated an excellent reliability (0.90). Item analysis indicated that dropping additional items would not have improved reliability. The test–retest reliability suggests stability over time (Spearman’s r = 0.83; 95% CL: 0.77, 0.88).

The final version of the WHO QualityRights Knowledge Questionnaire had 24 items (see **Table 1**). The minimum score obtained by participants was 5 and the maximum was 24, with a mean of 12.81 ± 4.76 and a median of 12.

### WHO QualityRights attitudes questionnaire

A total of 320 participants completed the WHO QualityRights Attitudes questionnaire. The total sample (i.e., the 320 participants that completed at least some of the survey questionnaires from the WHO QR questionnaires) coincides with the sample of completers of the WHO QR Attitudes questionnaire.

#### Validity

**Supplementary Table 7** shows the content validity assessment performed by the seven experts in human rights in mental health on the items’ consistency, clarity, difficulty, and worthiness to be included in the questionnaire. The maximum score for each item was 28. When evaluated for the performance of its items on these four dimensions (item consistency with the content area, item wording clarity, items perceived difficulty, and whether the items should be included in instrument), the questionnaire obtained an overall score of 459/476 and an average content validity index of 0.96, indicating that the WHO QR Attitudes items were relevant and clear.

**Supplementary Table 8** shows the face validity assessment performed by seven lay stakeholders. The first column reports the evaluation of the items’ clarity, the second describes if the items were easy to understand, whereas the third reports if the item was deemed relevant. The maximum score for each item was 21. The overall score of the questionnaire was 342/357, and all the items received high scores (ranging from 18 to 21), indicating that they were clear, easy to understand, and relevant for the population of interest.

**Figure 2:**
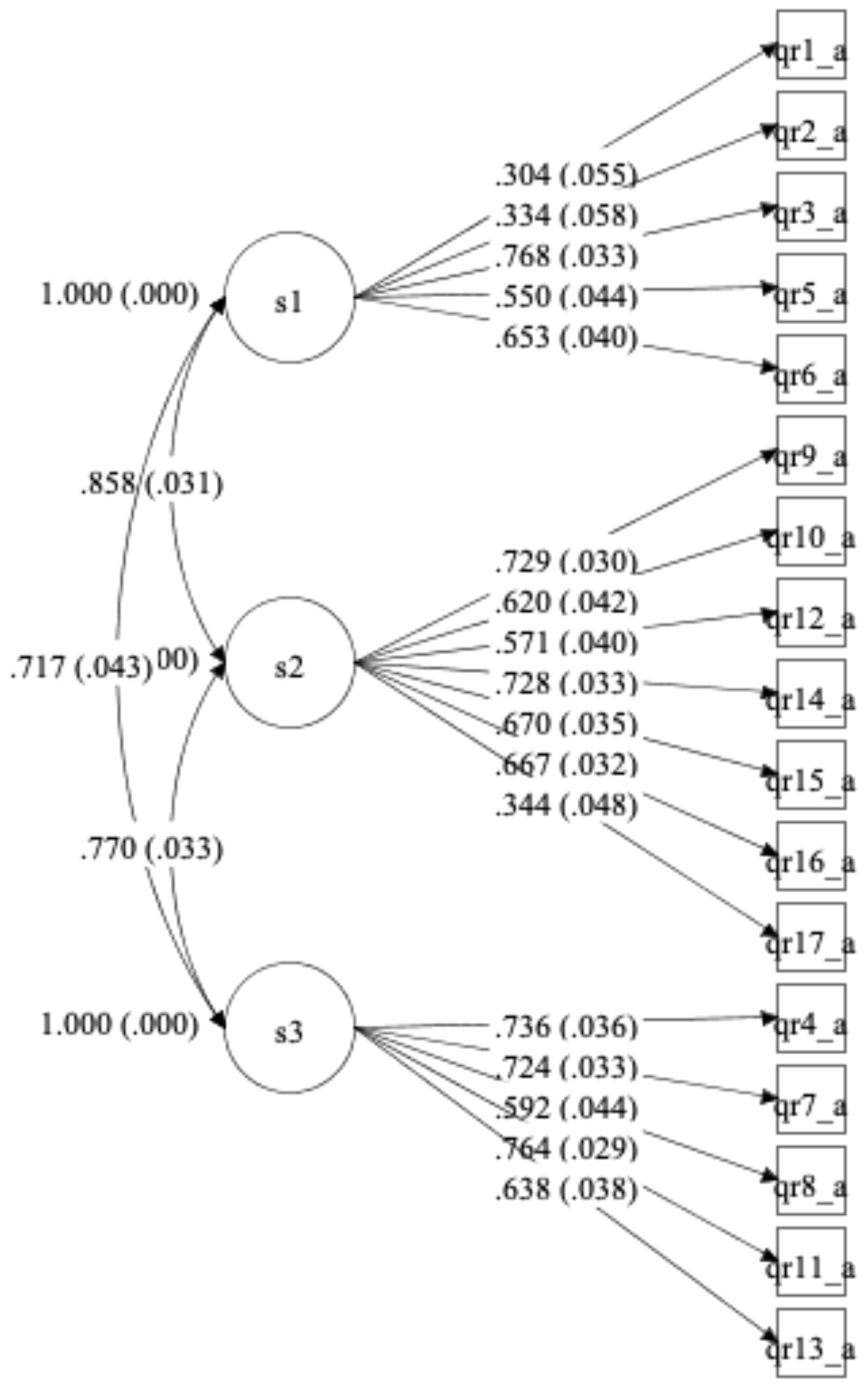
Confirmatory factor analysis path diagram for WHO QR Attitudes (final version). *Standardized loadings (and standard errors).

As shown in **Figure 2**, we conducted a Confirmatory Factor Analysis, fitting a model with the three factors postulated (attitudes toward mental health services approach, attitudes toward involuntary and coercive practices, and attitudes toward people with psychosocial disabilities or mental health conditions as decision-makers and full members of society). The model showed an acceptable fit (RMSEA = 0.074; CFI = 0.94; TLI = 0.93), and all the factor loadings were supported, with values greater than 0.3 and significant, indicating that our assessment results are in agreement with the conceptual model and the relationships that we postulated for the construct of interest.

Convergent and divergent validity evidence is presented in **Tables 4**, **5**. The results showed a moderate positive correlation between the WHO QR Attitudes questionnaire total score (whose higher score indicates negative attitudes) and the scores on the CAMI Authoritarianism and Social restrictiveness scales, a moderate negative correlation between the WHO QR Attitudes total score and the score at the CAMI Community mental health ideology scale, and a low negative correlation between the WHO QR Attitudes and the CAMI Benevolence subscale. The results are in the direction we had hypothesized.

**Table 4:** Evaluation of convergent and divergent validity between WHO QualityRights Attitudes and Community Attitudes toward the Mentally III subscales (n = 261).

	Total score QR	CAMI Authoritarianism	CAMI Benevolence	CAMI Social restrictiveness	CAMI Community ideology
**Total score QR**	1.00	0.68 95% CL (0.60, 0.74)	−0.38 95% CL (−0.48, −0.27)	0.60 95% CL (0.51, 0.67)	−0.54 95% CL (−0.61, −0.44)

**Table 5:** Evaluation of convergent and divergent validity between WHO QualityRights Attitudes and Attribution Questionnaire subscales (n = 242).

(n = 242)	Total score QR	AQ Blame	AQ Anger	AQ Pity	AQ Help	AQ Dangerousness	AQ Fear	AQ Avoidance	AQ Segregation	AQ Coercion
Total score QR	1.00	0.16 95% CL (0.03, 0.28)	0.29 95% CL (0.17, 0.40)	0.31 95% CL (0.19, 0.42)	−0.06 95% CL (−0.19, 0.06)	0.40 95% CL (0.29, 0.51)	0.41 95% CL (0.03, 0.28)	0.30 95% CL (0.17, 0.41)	0.41 95% CL (0.30, 0.51)	0.57 95% CL (0.48, 0.65)

Similarly, the results showed a moderate positive correlation between the WHO QR Attitudes total score and the scores on the Attribution Questionnaire Coercion and Segregation subscales, and a low positive correlation between the QR questionnaire total score and the scores on the Attribution Questionnaire Dangerousness, Fear, Avoidance, and Anger subscales. A low positive correlation was found between the WHO QR Attitudes total score and the score at the Attribution Questionnaire Pity subscale. Attribution Questionnaire Blame and Help subscales correlation were negligible. The results also are in the direction we had hypothesized.

#### Reliability

The overall Cronbach’s alpha value for the total scale indicated a good reliability (0.86). The internal consistency of the subscales was moderate or good (Cronbach’s alpha of 0.61 for Subscale 1, 0.77 for Subscale 2, and 0.75 for Subscale 3). Item analysis indicated that dropping additional items would not have improved reliability. The test–retest reliability suggests stability over time (Pearson’s r = 0.84; 95% CL, 0.78, 0.89).

The final version of the WHO QualityRights Attitudes Questionnaire coincides with the original version (see **Table 2**) and has 17 items. The minimum score obtained by participants was 17 and the maximum was 76, with a mean of 43.51 ± 11.25 and a median of 45.

### WHO QualityRights practices questionnaire

Eighty-nine participants reported that they worked in a mental health facility and all of them were asked to complete the WHO QR Practices questionnaire. Among these, 81 participants completed the questionnaire.

There were no meaningful differences between the total sample of participants working in a mental health facility and completers of the WHO QR Practices questionnaire regarding median age (median age was 34 both in the total sample of participants working in a mental health facility and among WHO QR Practices completers). There were slight differences regarding gender (in the total sample of participants working in a mental health facility, women were 51.7%, men were 47.2%, and other gender were 1.1%, whereas, among WHO QR Practices completers, women were 54.3%, men were 44.5%, and other gender were 1.2%).

#### Validity

**Supplementary Table 9** shows that the WHO QR Practices obtained an overall score of 328/336 for the performance of its items on the four content validity dimensions (item consistency with the content area, item wording clarity, items perceived difficulty, and whether the items should be included in instrument). An average content validity index of 0.976 was calculated from the overall score, indicating that the WHO QR Practices items were relevant and clear.

**Supplementary Table 10** shows that the overall score of the questionnaire for face validity was 242/252. All the items received high scores (ranging from 18 to 21), indicating that the items were clear, easy to understand, and relevant.

**Figure 3:**
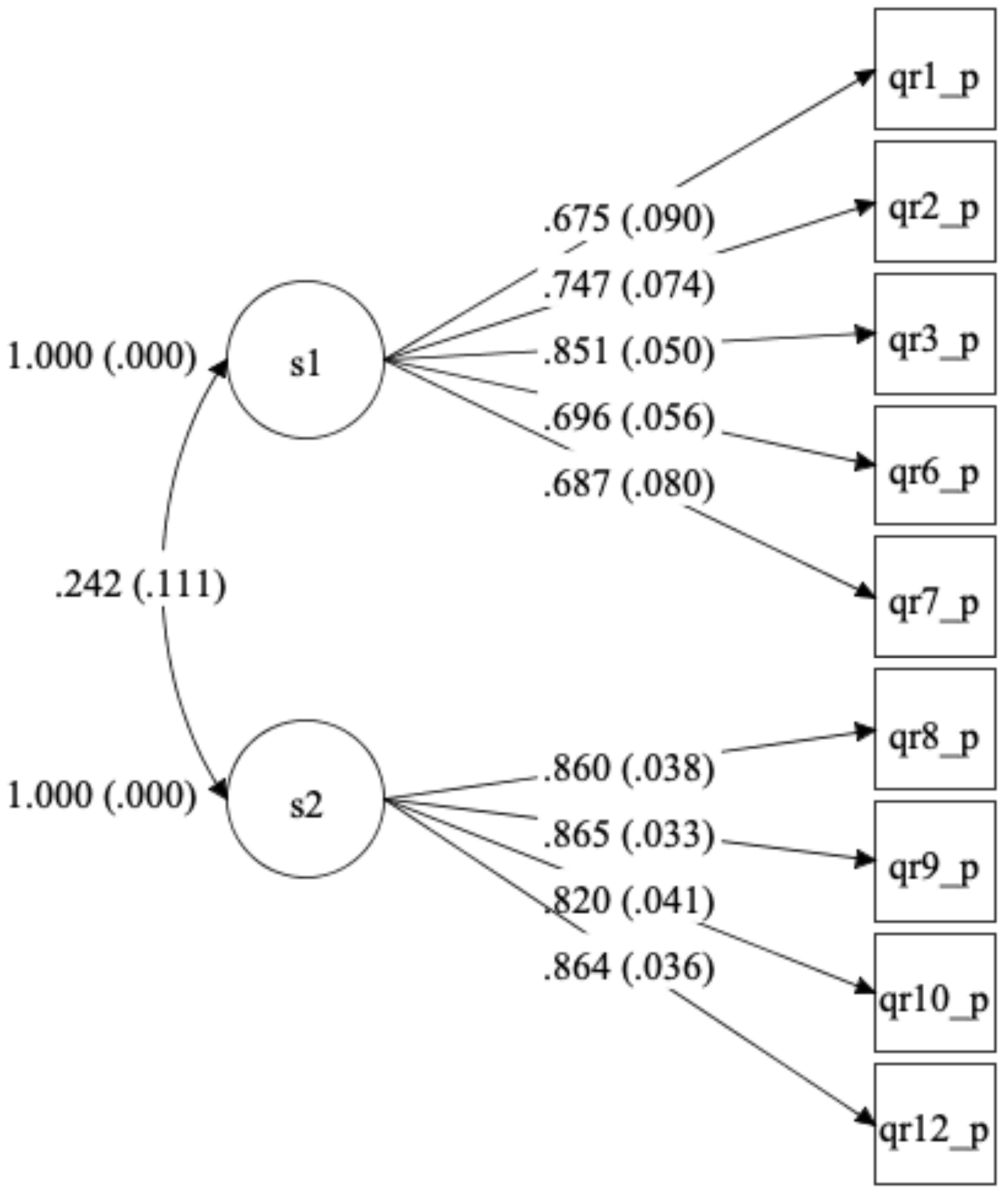
Confirmatory factor analysis path diagram for WHO QR Practices (final version). *Standardized loadings (and standard errors).

We conducted a Confirmatory Factor Analysis, fitting a model with the two factors postulated (practices related to substitute decision-making and coercion used by the respondent, and level of agreement—between the respondents and the mental health professionals working in their units—in the use of practices related to substitute decision-making and coercion). The only three items depicting positive practices (items 4, 5, and 11) had factor loadings with values lower than 0.3 and were excluded from the final model. The final nine-item model showed an acceptable fit (RMSEA = 0.070; CFI = 0.99; TLI = 0.99), and all the factor loadings were supported (see **Figure 3**), with values greater than 0.3 and significant, indicating that our assessment results are in agreement with the two factors conceptual model and the relationships we postulated for them.

#### Reliability

The Cronbach’s alpha value for the total scale indicated a good reliability (0.75). The internal consistency was moderate (Cronbach’s alpha of 0.74) for Subscale 1 and good for Subscale 2 (Cronbach’s alpha of 0.89). Item analysis indicated that dropping additional items would not have improved reliability.

Only 11 participants completed the questionnaire after 10 days, so it was not possible to evaluate the test–retest reliability. It is worth mentioning that data for the validation of the questionnaires were collected during a period when mental health workers were on strike and the stressful situation may have had a negative impact on their willingness to continue participating in the study.

The final version of the WHO QualityRights Practices Questionnaire had nine items: subscale A had five items, and subscale B had four items (See **Table 3**). For subscale A, the minimum score obtained by participants was 5 and the maximum was 26, with a mean of 9.33 ± 4.99 and a median of 7. For subscale B, the minimum score obtained by participants was 4 and the maximum was 20, with a mean of 11.22 ± 4.12 and a median of 12.

## Discussion

This study presents three recently designed questionnaires: the World Health Organization’s QualityRights Knowledge questionnaire (WHO QR Knowledge), the World Health Organization’s QualityRights Attitudes questionnaire (WHO QR Attitudes), and the World Health Organization’s QualityRights Practices questionnaire (WHO QR Practices). The analyses conducted indicate that the three questionnaires are valid and reliable instruments to evaluate the knowledge about the rights of persons with mental health conditions and psychosocial disabilities, the attitudes toward their role as rights holders, and mental health professionals’ practices related to substitute decision-making and coercion.

According to the experts engaged, the questionnaires showed excellent content validity. Their items were found to be consistent with the content area, clear, not difficult to understand, and deserving to be included in the instruments. Only one item of the WHO QR Knowledge questionnaire was deemed inadequate and removed. Face validity was also found to be high, with stakeholders from the general population reporting that the items were clear, not difficult, and worthy of being included in the questionnaires. This indicates that the questionnaires contain clear and relevant questions, that capture the domains of interest.

As postulated, the results from the CFAs indicated that an adequate one-factor model should be favored for the WHO QR Knowledge, a three-factor model for the WHO QR Attitudes, and a two-factor model for the WHO QR Practices. For the WHO QR Knowledge, the CFA revealed that 12 items did not contribute adequately to the scale, and, consequently, they were removed. All the other items contributed significantly to the scale, whose construct validity was adequate. The CFA of the WHO QR Attitudes revealed that every item in each of the three subscales (i.e., attitudes toward mental health services approach, attitudes toward involuntary and coercive practices, and attitudes toward people with psychosocial disabilities and mental health conditions as decision-makers and full members of society) contributed significantly to the respective subscales dimension and that the construct validity of each subscale was adequate. For the WHO QR Practices, the CFA revealed that the three items describing positive practices did not contribute adequately to their corresponding subscale; thus, they were removed. However, all other items in each of the two subscales (practices related to substitute decision-making and coercion used by the respondent, and level of agreement—between the respondents and the mental health professionals working in their units—in the use of practices related to substitute decision-making and coercion) contributed significantly to the respective subscales dimension, showing that their construct validity was adequate.

Additionally, the WHO QR Knowledge was able to differentiate between experts in human rights in mental health and non-experts. This supported the known group validity of the questionnaire, indicating that this instrument can be used to measure the construct of interest (i.e., knowledge about the rights of persons with mental health conditions and psychosocial disabilities).

Furthermore, for the WHO QR Attitudes, convergent validity estimates support that this questionnaire does measure what it is supposed to. As expected, high scores on the WHO QR Attitudes, which indicate negative attitudes, were positively correlated with high scores on the Authoritarianism and Social restrictiveness subscales of the CAMI, which reflect a view of persons with mental health conditions and psychosocial disabilities as a threat to society requiring coercive handling. Conversely, high scores on the WHO QR Attitudes were negatively correlated to high scores on the Benevolence and Community mental health ideology subscales of the CAMI, which reflect, respectively, a sympathetic view of persons with mental health conditions and psychosocial disabilities, based on humanistic principles, and the acceptance of their inclusion in the community. Similarly, high scores on the WHO QR Attitudes were positively correlated to high scores on the AQ subscales that indicate endorsement of negative stereotypes and discriminatory practices (i.e., Coercion, Segregation, Dangerousness, Fear, Avoidance, and Anger).

Interestingly, high scores on the WHO QR Attitudes were also positively correlated to the subscale indicating endorsement of Pity, considered in the AQ and other attitudes scales, as well as more generally, a “benevolent feeling” of sympathy toward people facing challenges due to their mental health condition and psychosocial disability. This understanding of disability was the tenet of outdated models of disability, such as the medical and the charity models, according to which people with disabilities have an impairment and, for this reason, they should be objects of pity and receive charitable resources for support. The WHO QR Attitudes was developed in line with the human rights–based approach of the CRPD, which considers people with disabilities as active and valuable members of society and not passive recipients of charity and objects of pity. The positive correlation between high scores on the WHO QR Attitudes and high scores on the AQ Pity subscale further demonstrates that the WHO QR Attitudes is different from previous scales in that its evaluation of attitudes is in line with the human rights–based approach.

The internal consistency for the three questionnaires was good, with overall Cronbach’s alpha values for the total scales of 0.90, 0.86, and 0.75 for the WHO QR Knowledge, the WHO QR Attitudes, and the WHO QR Practices, respectively. The internal consistency of the questionnaires subscales was also moderate or good, with Cronbach’s alpha values ranging from 0.61 to 0.89. The test–retest reliability suggested that both the WHO QR Knowledge and the WHO QR Attitudes are stable over time, whereas it was impossible to evaluate the long-term reliability of the WHO QR Practices because only a few mental health professionals completed this questionnaire during the re-assessment.

This study has several strengths. First, it is the first study to rigorously develop and investigate the psychometric properties of three instruments that follow the human rights approach and language promoted by the United Nations Convention on the Rights of Persons with Disabilities. Content, face, and construct validation methods and reliability testing were conducted to ensure these instruments were valid and reliable in evaluating the dimensions of interest. Second, persons with mental health conditions and psychosocial disabilities were involved in all the instruments’ development and validation phases, in line with the UN CRPD requirements. Third, to our knowledge, the WHO QR Knowledge and the WHO QR Practices are the first validated instruments to specifically evaluate the knowledge regarding persons with mental health conditions and psychosocial disabilities’ human rights as listed in the UN CRPD and the practices related to substitute decision-making and coercion, respectively. Similarly, the WHO QR Attitudes is the first validated instrument to focus specifically on assessing attitudes toward people with mental health conditions and psychosocial disabilities’ role as rights-holders.

Some limitations should also be considered in interpreting the results of this study. First, a convenience sample was used, formed by people from Ghana and it was not possible to collect information on people who refused to participate in the study. This might impose limitations on the generalizability of the findings. A second potential limitation is related to self-reporting and the possibility that participants may have underreported negative attitudes and practices related to substitute decision-making and coercion due to social desirability bias. Third, the test–retest reliability of the WHO QR Practices could not be evaluated due to the limited number of respondents at reassessment. Forth, we used Cronbach’s alpha to assess internal consistency. Although Cronbach’s alpha is a widely used measure for this purpose, it assumes that all items on the scale have equal factor loadings (or are tau-equivalent). When this is not the case, the use of Cronbach’s alpha may lead to an inaccurate estimate of internal consistency. Future studies should be conducted with representative samples and different populations to check if the findings from this research are generalizable. Measures of social desirability such as the Marlowe–Crowne Social Desirability Scale could be used to evaluate if participants may have underreported negative attitudes and practices. The stability over time of the WHO QR Practices should be investigated in a study targeting mental health professionals at recruitment. Furthermore, additional approaches could be used to evaluate internal consistency (e.g., congeneric measurement models).

The World Health Organization’s QualityRights Knowledge questionnaire (WHO QR Knowledge), the World Health Organization’s QualityRights Attitudes questionnaire (WHO QR Attitudes), and the World Health Organization’s QualityRights Practices questionnaire (WHO QR Practices) displayed excellent reliability and validity. This finding lends support to their use both within mental health services and in the general population for a better understanding of current knowledge, attitudes, and practices related to a human rights–based approach to mental health in mental health services and the community.

## Data Availability

The raw data supporting the conclusions of this article will be made available by the authors upon reasonable request. Requests to access the datasets should be directed to MFM, mfmoro@gmail.com.
