# Supplementary material for: Evaluating the psychometric properties of three WHO instruments to assess knowledge about human rights, attitudes toward persons with mental health conditions and psychosocial disabilities, and practices related to substitute decision-making and coercion in mental health
Source: Front Psychiatry. 2024 Sep 6;15:1435608. doi: 10.3389/fpsyt.2024.1435608 (PMC11413867; doi:10.3389/fpsyt.2024.1435608)
Supplement: Supplementary file 1 [file Table1.docx]

Supplementary Table 1: World Health Organization’s QualityRights Knowledge questionnaire (WHO QR Knowledge) – Original version

| **Please indicate if the following statements are True (T) or False (F)** | | | |
| --- | --- | --- | --- |
|  | | **T** | **F** |
| **1** | All 30 human rights within the Universal Declaration of Human Rights (UDHR) are needed to live a good life. |  |  |
| **2** | The Universal Declaration of Human Rights (UDHR) is a law. |  |  |
| **3** | Human rights can never be restricted. |  |  |
| **4** | Violations of human rights can only be carried out by individuals, not by governments. |  |  |
| **5** | The Convention on the Rights of People with Disabilities (CRPD) is a convention that protects the rights of all marginalized groups. |  |  |
| **6** | According to the CRPD, people with dementia have the right to live in the community and to choose their living arrangements |  |  |
| **7** | Informed consent is when a person’s family members receive information about different possible treatment options in order to make an informed decision. |  |  |
| **8** | Advance plans/directives are documents made by health practitioners to plan in advance the treatment of people using the service. |  |  |
| **9** | To promote legal capacity, family members, caregivers, and supporters should help people make decisions by explaining different options but should not assist in communicating decisions to others. |  |  |
| The Convention on the Rights of People with Disabilities: | | | |
| **10** | Was intended to create new rights for people with disabilities |  |  |
| **11** | Adopts the medical and charity models of disability |  |  |
| **12** | Adopts the social and human rights models of disability |  |  |
| **13** | Is binding on countries which have ratified it |  |  |
| According to the human rights model, people diagnosed, perceived or self-identifying as having a mental health condition, psychosocial, intellectual or cognitive disability: | | | |
| **14** | Must show their ability to understand rights in order to claim them |  |  |
| **15** | Have the right to have attitudinal and environmental barriers removed |  |  |
| **16** | Cannot fully participate in society on an equal basis with others because of attitudinal and environmental barriers |  |  |
| **17** | Need treatment to 'fix' or heal them |  |  |
| The right to liberty and security of a person in the CRPD means that: | | | |
| **18** | People cannot be detained based on the fact that they have a disability |  |  |
| **19** | People can be detained because they have a disability as long as other criteria such as dangerousness or medical necessity are also met |  |  |
| **20** | People with disabilities can only be deprived of liberty for the same reasons as any other person |  |  |
| **21** | Mental health laws can authorise people to be detained if they are diagnosed with a mental health condition and if they are perceived as dangerous |  |  |
| In supported decision-making, support: | | | |
| **22** | Can be declined by the person |  |  |
| **23** | Requirements may vary throughout life |  |  |
| **24** | Should concern only complex decisions |  |  |
| **25** | Should increase throughout life |  |  |
| Forced treatment, seclusion, and restraint: | | | |
| **26** | Keep people safe |  |  |
| **27** | Are forms of coercion |  |  |
| **28** | Improve recovery if used correctly |  |  |
| **29** | Can cause harm |  |  |
| Which of the following promote the right to legal capacity? | | | |
| **30** | User –led recovery and/or treatment plans |  |  |
| **31** | Healthcare provider-led recovery and/or treatment plans |  |  |
| **32** | Informed consent |  |  |
| **33** | Substitute-decision making |  |  |
| According to the Convention on the Rights of Persons with Disabilities: | | | |
| **34** | Governments must make sure that people with disabilities are free from torture or cruel, inhuman or degrading treatment or punishment |  |  |
| **35** | The use of restraints is only allowed in cases where the service lacks adequate human resources |  |  |
| **36** | Countries must make laws to ensure that people with disabilities are protected from exploitation, violence and abuse in the home and in the community |  |  |
| **37** | There is no need to make laws to protect people with disabilities from exploitation, violence and abuse because the CRPD already ensures their protection |  |  |

Supplementary Table 2: World Health Organization’s QualityRights Attitudes questionnaire (WHO QR Attitudes) Subscales 1, 2, and 3 – Final version

| Subscale 1 - Attitudes towards mental health services approach | |
| --- | --- |
| 1 | I am quick Nothing can be improved within mental health services without additional resources |
| 2 | The service environment has little to do with people's mental health and well-being |
| 3 | People with dementia should always live in group homes where staff can take care of them |
| 5 | Taking medication is the most important factor to help people with mental health conditions get better |
| 6 | You can only inspire hope once a person is no longer experiencing symptoms |
| Subscale 2 - Attitudes towards involuntary and coercive practices | |
| 9 | The opinions of health practitioners about care and treatment should carry more weight than those of a person with an intellectual disability |
| 10 | It is acceptable to pressure people using mental health services to take treatment that they don't want |
| 12 | When people experience a crisis, health practitioners or families should make decisions based on their ideas about what is best for them |
| 14 | Controlling people using mental health services is necessary to maintain order |
| 15 | The use of seclusion and restraint is needed if people using mental health services become threatening |
| 16 | People at risk of harming themselves or others should be isolated in a locked room |
| 17 | Involuntary admission does more harm than good |
| Subscale 3 - Attitudes towards people with psychosocial disabilities or mental health conditions as decision-makers  and full members of society | |
| 4 | People with psychosocial disabilities/mental health conditions should not be hired in work requiring direct contact with the public |
| 7 | People using mental health services should be empowered to make their own decisions about their treatment |
| 8 | Following advice of other people who have experienced mental health issues is too risky |
| 11 | Persons with mental health conditions should not be given important responsibilities |
| 13 | People with intellectual disabilities have the right to make their own decisions, even if I don’t agree with them |

Supplementary Table 3: World Health Organization’s QualityRights Practices questionnaire (Sub-scales 1 and 2) – Original version

| Please indicate how many times in the last month you used the following strategies **within your psychiatric unit**.  Select only one option for each statement. | | | | | | | | |
| --- | --- | --- | --- | --- | --- | --- | --- | --- |
|  | | Every day | A few times a week | Once a week | A few times a month | Once a month or less | A few times in the last three months | Never |
| 1 | **I used seclusion**  (for instance, ordering or keeping service users in a locked room) | 7 |  |  |  |  |  | 1 |
| 2 | **I used physical restraints**  (for instance, using ties or other mechanical devices to restrain service users) |  |  |  |  |  |  |  |
| 3 | **I prescribed or administered a treatment although the service user did not want it** |  |  |  |  |  |  |  |
| 4 | **I supported service users to write their recovery plan** |  |  |  |  |  |  |  |
| 5 | **I informed service users about their rights**  (including their right to refuse treatment) |  |  |  |  |  |  |  |
| 6 | **I used chemical restraints**  (for instance, prescribing or administering an injection to calm the behavior of service users without their consent) |  |  |  |  |  |  |  |
| 7 | **I yelled or used verbal aggression to get service users to comply with requests** |  |  |  |  |  |  |  |

| For each statement, mark the box that most accurately reflects your response. | | | | | | |
| --- | --- | --- | --- | --- | --- | --- |
|  |  | A lot less than me | Less  than me | As much as me | More than me | A lot more than me |
| 8 | Mental health professionals in my unit use seclusion and physical/chemical restraints | 1 |  |  |  | 5 |
| 9 | Mental health professionals in my unit yell or use verbal aggression to get service users to comply with requests |  |  |  |  |  |
| 10 | Mental health professionals in my unit prescribe or administer treatments to control the behavior of service users |  |  |  |  |  |
| 11 | Mental health professionals in my unit respect the will and preferences of service users |  |  |  |  |  |
| 12 | Mental health professionals in my unit use restraints to control unsettled situations in the ward |  |  |  |  |  |

Supplementary Table 4: Sociodemographic characteristic of the total sample recruited for the evaluation of the psychometric properties of the WHO QualityRights questionnaires.

|  | **Frequency** | **Percent** |
| --- | --- | --- |
| **Gender** |  |  |
| Women | 127 | 39.69 |
| Men | 191 | 59.69 |
| Other gender | 2 | 0.63 |
| **Age** |  |  |
| 18-24 | 3 | 0.94 |
| 25-34 | 122 | 38.13 |
| 35-44 | 116 | 36.25 |
| 45-54 | 41 | 12.81 |
| 55-64 | 32 | 10.00 |
| 65 and older | 6 | 1.88 |
| **Education** |  |  |
| Less than high school degree | 3 | 0.94 |
| High school degree or equivalent | 32 | 10.00 |
| Vocational/Technical School (2 years) | 4 | 1.25 |
| Some College | 32 | 10.00 |
| College Graduate (4 year) | 90 | 28.13 |
| Master's Degree | 89 | 27.81 |
| Doctoral Degree (PhD) | 6 | 1.88 |
| Professional Degree (MD,JD, etc.) | 25 | 7.81 |
| Other | 39 | 12.19 |
| **Background** |  |  |
| Person with psychosocial, intellectual, and cognitive disability or mental health condition | 51 | 15.94 |
| Person with other disabilities | 48 | 15.00 |
| Family member or care partner | 13 | 4.06 |
| Mental health or related practitioner | 88 | 27.50 |
| Health practitioner | 57 | 17.81 |
| Lawyer | 4 | 1.25 |
| Human rights advocate | 35 | 10.94 |
| Policy Maker / Analyst | 5 | 1.56 |
| Academia | 8 | 2.50 |
| Other | 11 | 3.44 |
| **Language** |  |  |
| Akan (Fante, Asante Twi and Akuapem Twi) | 92 | 28.75 |
| Akposo | 0 | 0.00 |
| Dagaare | 1 | 0.31 |
| Dagbani | 6 | 1.88 |
| Dangme | 2 | 0.63 |
| English | 130 | 40.63 |
| Ewe | 25 | 7.81 |
| Ga | 39 | 12.19 |
| Gonja | 4 | 1.25 |
| Kasem | 1 | 0.31 |
| Nzema | 1 | 0.31 |
| Other | 19 | 5.94 |
| **Region** |  |  |
| Ahafo Region | 3 | 0.94 |
| Ashanti | 21 | 6.56 |
| Bono East Region | 2 | 0.63 |
| Bono Region | 8 | 2.50 |
| Central | 36 | 11.25 |
| Eastern | 10 | 3.13 |
| Greater Accra | 165 | 51.56 |
| North East | 0 | 0.00 |
| Northern | 13 | 4.06 |
| Oti | 11 | 3.44 |
| Savannah | 4 | 1.25 |
| Upper East | 14 | 4.38 |
| Upper West | 3 | 0.94 |
| Volta Region | 14 | 4.38 |
| Western North | 2 | 0.63 |
| Western Region | 14 | 4.38 |
| **Person with a psychosocial disability or mental health condition in the close family** |  |  |
| Yes | 113 | 35.31 |
| No | 207 | 64.69 |
| **Participant identifies as a person with a psychosocial disability or mental health condition** |  |  |
| Yes | 61 | 19.06 |
| No | 259 | 80.94 |
|  | **320** | **100.00** |

Supplementary Table 5: Content validity of WHO QualityRights Knowledge

| **Items** | **Consistency** | **Clarity** | **Difficulty** | **Inclusion** | **Total score** |
| --- | --- | --- | --- | --- | --- |
| **QR1_K** | 6/7 | 7/7 | 6/7 | 7/7 | 26/28 |
| **QR2_K** | 6/7 | 6/7 | 6/7 | 7/7 | 25/28 |
| **QR3_K** | 7/7 | 7/7 | 7/7 | 7/7 | 28/28 |
| **QR4_K** | 6/7 | 6/7 | 6/7 | 7/7 | 25/28 |
| **QR5_K** | 7/7 | 7/7 | 7/7 | 7/7 | 28/28 |
| **QR6_K** | 7/7 | 7/7 | 7/7 | 7/7 | 28/28 |
| **QR7_K** | 7/7 | 7/7 | 7/7 | 7/7 | 28/28 |
| **QR8_K** | 7/7 | 6/7 | 7/7 | 7/7 | 27/28 |
| **QR9_K** | 7/7 | 7/7 | 7/7 | 7/7 | 28/28 |
| **QR10_K** | 6/7 | 6/7 | 7/7 | 7/7 | 26/28 |
| **QR11_K** | 6/7 | 6/7 | 7/7 | 6/7 | 25/28 |
| **QR12_K** | 7/7 | 7/7 | 7/7 | 7/7 | 28/28 |
| **QR13_K** | 7/7 | 5/7 | 7/7 | 7/7 | 26/28 |
| **QR14_K** | 6/7 | 6/7 | 7/7 | 7/7 | 26/28 |
| **QR15_K** | 7/7 | 7/7 | 7/7 | 7/7 | 28/28 |
| **QR16_K** | 7/7 | 7/7 | 7/7 | 7/7 | 28/28 |
| **QR17_K** | 5/7 | 6/7 | 6/7 | 7/7 | 24/28 |
| **QR18_K** | 5/7 | 6/7 | 6/7 | 6/7 | 23/28 |
| **QR19_K** | 7/7 | 5/7 | 3/7 | 3/7 | 18/28 |
| **QR20_K** | 5/7 | 6/7 | 6/7 | 7/7 | 24/28 |
| **QR21_K** | 6/7 | 7/7 | 7/7 | 7/7 | 27/28 |
| **QR22_K** | 7/7 | 6/7 | 6/7 | 7/7 | 26/28 |
| **QR23_K** | 7/7 | 7/7 | 7/7 | 7/7 | 28/28 |
| **QR24_K** | 5/7 | 6/7 | 7/7 | 7/7 | 25/28 |
| **QR25_K** | 6/7 | 5/7 | 5/7 | 7/7 | 23/28 |
| **QR26_K** | 5/7 | 7/7 | 6/7 | 6/7 | 24/28 |
| **QR27_K** | 7/7 | 7/7 | 7/7 | 7/7 | 28/28 |
| **QR28_K** | 5/7 | 6/7 | 6/7 | 6/7 | 23/28 |
| **QR29_K** | 7/7 | 7/7 | 6/7 | 7/7 | 27/28 |
| **QR30_K** | 7/7 | 7/7 | 7/7 | 7/7 | 28/28 |
| **QR31_K** | 6/7 | 6/7 | 5/7 | 7/7 | 24/28 |
| **QR32_K** | 7/7 | 7/7 | 7/7 | 7/7 | 28/28 |
| **QR33_K** | 7/7 | 6/7 | 6/7 | 7/7 | 26/28 |
| **QR34_K** | 7/7 | 7/7 | 7/7 | 7/7 | 28/28 |
| **QR35_K** | 5/7 | 6/7 | 7/7 | 7/7 | 25/28 |
| **QR36_K** | 7/7 | 7/7 | 7/7 | 7/7 | 28/28 |
| **QR37_K** | 6/7 | 6/7 | 6/7 | 6/7 | 24/28 |
|  | 235/259 | 237/259 | 239/259 | 250/259 | 961/1,036 |
| **Content Validity Index** |  |  |  |  | **0.928** |

Supplementary Table 6: Face validity of WHO QualityRights Knowledge

| **Items** | **Clarity** | **Easiness to understand** | **Relevance** | **Total score** |
| --- | --- | --- | --- | --- |
| **QR1_K** | 7/7 | 7/7 | 7/7 | 21/21 |
| **QR2_K** | 6/7 | 7/7 | 6/7 | 19/21 |
| **QR3_K** | 6/7 | 7/7 | 6/7 | 19/21 |
| **QR4_K** | 6/7 | 7/7 | 6/7 | 19/21 |
| **QR5_K** | 7/7 | 7/7 | 7/7 | 21/21 |
| **QR6_K** | 7/7 | 7/7 | 7/7 | 21/21 |
| **QR7_K** | 6/7 | 7/7 | 6/7 | 19/21 |
| **QR8_K** | 7/7 | 7/7 | 6/7 | 20/21 |
| **QR9_K** | 6/7 | 7/7 | 6/7 | 19/21 |
| **QR10_K** | 6/7 | 7/7 | 5/7 | 18/21 |
| **QR11_K** | 7/7 | 7/7 | 7/7 | 21/21 |
| **QR12_K** | 7/7 | 7/7 | 7/7 | 21/21 |
| **QR13_K** | 6/7 | 7/7 | 6/7 | 19/21 |
| **QR14_K** | 6/7 | 7/7 | 6/7 | 19/21 |
| **QR15_K** | 7/7 | 7/7 | 7/7 | 21/21 |
| **QR16_K** | 6/7 | 7/7 | 6/7 | 19/21 |
| **QR17_K** | 6/7 | 7/7 | 6/7 | 19/21 |
| **QR18_K** | 5/7 | 6/7 | 5/7 | 16/21 |
| **QR19_K*** | --- | --- | --- | --- |
| **QR20_K** | 6/7 | 6/7 | 6/7 | 18/21 |
| **QR21_K** | 5/7 | 6/7 | 6/7 | 17/21 |
| **QR22_K** | 7/7 | 7/7 | 7/7 | 21/21 |
| **QR23_K** | 7/7 | 7/7 | 7/7 | 21/21 |
| **QR24_K** | 5/7 | 6/7 | 6/7 | 17/21 |
| **QR25_K** | 6/7 | 7/7 | 6/7 | 19/21 |
| **QR26_K** | 6/7 | 7/7 | 6/7 | 19/21 |
| **QR27_K** | 6/7 | 7/7 | 6/7 | 19/21 |
| **QR28_K** | 6/7 | 7/7 | 6/7 | 19/21 |
| **QR29_K** | 7/7 | 7/7 | 6/7 | 20/21 |
| **QR30_K** | 6/7 | 6/7 | 7/7 | 19/21 |
| **QR31_K** | 6/7 | 7/7 | 6/7 | 19/21 |
| **QR32_K** | 7/7 | 7/7 | 7/7 | 21/21 |
| **QR33_K** | 7/7 | 7/7 | 7/7 | 21/21 |
| **QR34_K** | 7/7 | 7/7 | 7/7 | 21/21 |
| **QR35_K** | 6/7 | 7/7 | 6/7 | 19/21 |
| **QR36_K** | 7/7 | 7/7 | 7/7 | 21/21 |
| **QR37_K** | 6/7 | 7/7 | 6/7 | 19/21 |
|  | 227/252 | 247/252 | 227/252 | 701/756 |

*Item eliminated based on content validity evaluation

Supplementary Table 7: Content Validity Index of WHO QualityRights Attitudes

| **Items** | **Consistency** | **Clarity** | **Difficulty** | **Inclusion** | **Total score** |
| --- | --- | --- | --- | --- | --- |
| **QR1_A** | 7/7 | 7/7 | 6/7 | 7/7 | 27/28 |
| **QR1_B** | 6/7 | 6/7 | 6/7 | 6/7 | 24/28 |
| **QR1_C** | 7/7 | 6/7 | 7/7 | 6/7 | 26/28 |
| **QR1_D** | 7/7 | 7/7 | 7/7 | 7/7 | 28/28 |
| **QR1_E** | 7/7 | 7/7 | 7/7 | 6/7 | 27/28 |
| **QR1_F** | 7/7 | 6/7 | 7/7 | 7/7 | 27/28 |
| **QR1_G** | 7/7 | 7/7 | 7/7 | 7/7 | 28/28 |
| **QR1_H** | 7/7 | 7/7 | 7/7 | 6/7 | 27/28 |
| **QR1_I** | 7/7 | 7/7 | 6/7 | 6/7 | 26/28 |
| **QR1_J** | 7/7 | 6/7 | 5/7 | 7/7 | 25/28 |
| **QR1_K** | 7/7 | 7/7 | 6/7 | 7/7 | 27/28 |
| **QR1_L** | 7/7 | 7/7 | 7/7 | 7/7 | 28/28 |
| **QR1_M** | 7/7 | 7/7 | 7/7 | 7/7 | 28/28 |
| **QR1_N** | 7/7 | 7/7 | 7/7 | 7/7 | 28/28 |
| **QR1_O** | 7/7 | 7/7 | 7/7 | 6/7 | 27/28 |
| **QR1_P** | 7/7 | 7/7 | 7/7 | 7/7 | 28/28 |
| **QR1_Q** | 7/7 | 7/7 | 7/7 | 7/7 | 28/28 |
|  | 118/119 | 115/119 | 113/119 | 113/119 | 459/476 |
| **Content**  **Validity Index** |  |  |  |  | **0.964** |

Supplementary Table 8: Face Validity of WHO QualityRights Attitudes

| **Items** | **Clarity** | **Easiness to understand** | **Relevance** | **Total score** |
| --- | --- | --- | --- | --- |
| **QR1_A** | 7/7 | 7/7 | 7/7 | 21/21 |
| **QR2_A** | 7/7 | 7/7 | 6/7 | 20/21 |
| **QR3_A** | 7/7 | 7/7 | 6/7 | 20/21 |
| **QR4_A** | 7/7 | 7/7 | 6/7 | 20/21 |
| **QR5_A** | 7/7 | 7/7 | 7/7 | 21/21 |
| **QR6_A** | 7/7 | 7/7 | 6/7 | 20/21 |
| **QR7_A** | 7/7 | 7/7 | 7/7 | 21/21 |
| **QR8_A** | 7/7 | 7/7 | 6/7 | 20/21 |
| **QR9_A** | 6/7 | 7/7 | 5/7 | 18/21 |
| **QR10_A** | 7/7 | 7/7 | 6/7 | 20/21 |
| **QR11_A** | 7/7 | 7/7 | 6/7 | 20/21 |
| **QR12_A** | 6/7 | 7/7 | 6/7 | 19/21 |
| **QR13_A** | 7/7 | 7/7 | 7/7 | 21/21 |
| **QR14_A** | 7/7 | 7/7 | 7/7 | 21/21 |
| **QR15_A** | 7/7 | 7/7 | 6/7 | 20/21 |
| **QR16_A** | 7/7 | 7/7 | 6/7 | 20/21 |
| **QR17_A** | 7/7 | 6/7 | 7/7 | 20/21 |
|  | 117/119 | 118/119 | 107/119 | 342/357 |

Supplementary Table 9: Content Validity Index of WHO QualityRights Practices

| **Items** | **Consistency** | **Clarity** | **Difficulty** | **Inclusion** | **Total score** |
| --- | --- | --- | --- | --- | --- |
| **QR1_P** | 7/7 | 7/7 | 7/7 | 7/7 | 28/28 |
| **QR2_P** | 6/7 | 7/7 | 7/7 | 7/7 | 27/28 |
| **QR3_P** | 7/7 | 6/7 | 7/7 | 7/7 | 27/28 |
| **QR4_P** | 7/7 | 7/7 | 7/7 | 7/7 | 28/28 |
| **QR5_P** | 7/7 | 7/7 | 7/7 | 7/7 | 28/28 |
| **QR6_P** | 7/7 | 7/7 | 7/7 | 7/7 | 28/28 |
| **QR7_P** | 7/7 | 7/7 | 7/7 | 7/7 | 28/28 |
| **QR8_P** | 6/7 | 6/7 | 7/7 | 6/7 | 25/28 |
| **QR9_P** | 7/7 | 7/7 | 7/7 | 7/7 | 28/28 |
| **QR10_P** | 7/7 | 7/7 | 7/7 | 6/7 | 27/28 |
| **QR11_P** | 7/7 | 7/7 | 6/7 | 7/7 | 27/28 |
| **QR12_P** | 6/7 | 7/7 | 7/7 | 7/7 | 27/28 |
|  | 81/84 | 82/84 | 83/84 | 82/84 | 328/336 |
| **Content**  **Validity Index** |  |  |  |  | **0.976** |

Supplementary Table 10: Face Validity of WHO QualityRights Practices

| **Items** | **Clarity** | **Easiness to understand** | **Relevance** | **Total score** |
| --- | --- | --- | --- | --- |
| **QR1_P** | 7/7 | 7/7 | 6/7 | 20/21 |
| **QR2_P** | 7/7 | 7/7 | 6/7 | 20/21 |
| **QR3_P** | 7/7 | 7/7 | 6/7 | 20/21 |
| **QR4_P** | 7/7 | 7/7 | 7/7 | 21/21 |
| **QR5_P** | 7/7 | 7/7 | 7/7 | 21/21 |
| **QR6_P** | 7/7 | 7/7 | 6/7 | 20/21 |
| **QR7_P** | 7/7 | 7/7 | 6/7 | 20/21 |
| **QR8_P** | 6/7 | 6/7 | 6/7 | 18/21 |
| **QR9_P** | 7/7 | 7/7 | 6/7 | 20/21 |
| **QR10_P** | 7/7 | 7/7 | 7/7 | 21/21 |
| **QR11_P** | 7/7 | 7/7 | 7/7 | 21/21 |
| **QR12_P** | 7/7 | 7/7 | 6/7 | 20/21 |
|  | 83/84 | 83/84 | 76/84 | 242/252 |
